# Strategies and effects of school-based interventions to promote active school transportation by bicycle among children and adolescents: a systematic review

**DOI:** 10.1186/s12966-020-01035-1

**Published:** 2020-11-12

**Authors:** Dorothea M. I. Schönbach, Teatske M. Altenburg, Adilson Marques, Mai J. M. Chinapaw, Yolanda Demetriou

**Affiliations:** 1 Department of Sport and Health Sciences, Technical University of Munich, Munich, Germany; 2 Amsterdam UMC, Vrije Universiteit Amsterdam, Department of Public and Occupational Health, Amsterdam Public Health Research Institute, Amsterdam, The Netherlands; 3 CIPER, Faculty of Human Kinetics, University of Lisbon, Lisbon, Portugal

**Keywords:** PRISMA, Program, Educational facilities, Pupil, Active school travel, Biking, (Randomized) controlled trial

## Abstract

**Background:**

Promoting cycling to school may benefit establishing a lifelong physical activity routine. This systematic review aimed to summarize the evidence on strategies and effects of school-based interventions focusing on increasing active school transport by bicycle.

**Methods:**

A literature search based on “PICo” was conducted in eight electronic databases. Randomized and non-randomized controlled trials with primary/secondary school students of all ages were included that conducted pre-post measurements of a school-based intervention aimed at promoting active school travel by bicycle and were published in English between 2000 and 2019. The methodological quality was assessed using the “Effective Public Health Practice Project” tool for quantitative studies. Applied behavior change techniques were identified using the “BCT Taxonomy v1”. Two independent researchers undertook the screening, data extraction, appraisal of study quality, and behavior change techniques.

**Results:**

Nine studies investigating seven unique interventions performed between 2012 and 2018 were included. All studies were rated as weak quality. The narrative synthesis identified 19 applied behavior change techniques clustered in eleven main groups according to their similarities and a variety of 35 different outcome variables classified into seven main groups. Most outcomes were related to active school travel and psychosocial factors, followed by physical fitness, physical activity levels, weight status, active travel and cycling skills. Four studies, examining in total nine different outcomes, found a significant effect in favor of the intervention group on bicycle trips to school (boys only), percentage of daily cycling trips to school, parental/child self-efficacy, parental outcome expectations, moderate-to-vigorous intensity physical activity (total, from cycling, before/after school), and total basic cycling skills. Seven of these outcomes were only examined in two studies conducting the same intervention in children, a voluntary bicycle train to/from school accompanied by adults, including the following clustered main groups of behavior change techniques: shaping knowledge, comparison of behavior, repetition and substitution as well as antecedents.

**Conclusions:**

The applied strategies in a bicycle train intervention among children indicated great potential to increase cycling to school. Our findings provide relevant insights for the design and implementation of future school-based interventions targeting active school transport by bicycle.

**Trial registration:**

This systematic review has been registered in the international prospective register of systematic reviews “PROSPERO” at (registration number: CRD42019125192).

**Supplementary information:**

**Supplementary information** accompanies this paper at 10.1186/s12966-020-01035-1.

## Background

There is increasing focus on identifying effective strategies to improve physical activity (PA) among children and adolescents [1]. Most young people in Europe do not achieve the recommended daily accumulation of 60 min (min) in moderate-to-vigorous intensity physical activity (MVPA) [2] of the World Health Organization (WHO) [3], in spite of the well-known health benefits [4]. Both PA-related health benefits, which can persist into adult life, and a variety of health problems in adulthood, including overweight or obesity [5], appear to have their origin in early life [5, 6]. Therefore, the low compliance with PA recommendations is alarming. Since PA habits are established early in life, promoting PA from an early age is required [7–11]. Active school travel (AST) is a source of habitual PA for students and therefore highly recommended [12]. AST is positively related to total daily PA [13–16], school day PA [14, 17] as well as PA before and after school [14, 15, 17]. Cycling is an important option for AST. In England, those who cycled for AST accumulated on average 1.4 h of cycling per week, which contributed 20% of recommended weekly PA [16]. As a result, a higher percentage of cyclists (36%) aged 5 to 15 years meet the weekly WHO recommendation of PA compared to walkers (25%) and those who did not walk or cycle to/from school (22%) [16]. In particular, adolescent girls, who have lower levels of PA [18] and perceive more barriers to PA (e.g., lack of energy) [19], may benefit more from participating in AST than adolescent boys [14]. Previous research showed that adolescent girls from New Zealand who participated in AST were more likely to meet the PA recommendations compared to passive travelers [14]. This was not the case for boys [14].

In addition, AST has been positively associated with body composition [15, 20], positive emotions [21], and cognitive performance (only in adolescent girls) [22]. Compared to walking, cycling is generally of higher intensity [23]. Thereby, AST by bicycle contributes to cardiovascular fitness [23] and may reduce the future risk of cardiovascular diseases. In addition, AST has been positively associated with environmental factors, such as reduction of traffic [24, 25] which contributes to a minimization of air pollution [23, 25] and enhancement of road safety [24]. Furthermore, adopting a daily AST routine including journeys to and from school [26] as early as possible may lead to a potentially lifelong habit of active transport (AT) [16] including journeys to any other destination. Moreover, a study in Ireland showed that AST by bicycle increases the mobility of adolescents living further away from school [27]. Bicycles are also the fastest means of transportation for distances less than 5 km in cities, especially when car traffic is congested [28].

Studies in Germany showed that most children and adolescents aged up to 17 years own a bicycle (57 to 98%) [29]. However, only 8% [30] to 22.2% [31] cycle to/from school daily or usually. Additionally, more boys (23.8%) than girls (20.6%) cycle to school in Germany [31]. In the Czech Republic, the percentages of boys (5.7, 3.2, 2.2%) and girls (2.3, 0.5, 2%) aged 11 to 15 years who cycled to/from school between 2006, 2010 and 2014 decreased over time [32]. According to these data from Germany and the Czech Republic, cycling is a less common form of AST, cycling habits differ by gender in favor of boys, and there might be a declining trend in some European countries.

Following this, researchers have increased interest in developing AST interventions in the last years [33]. A previous systematic review focused on the effects of AST interventions aiming to promote walking [24]. No previous systematic review dealt exclusively with the effectiveness of intervention strategies targeting cycling as means of AST, which is required for adequate policy decisions in this field [1]. Thus, the aims of this systematic review were to summarize the evidence on strategies and effects of (randomized) controlled interventions that promote cycling to school as a mode of AST among primary and/or secondary school students.

## Methods

The methodological procedure of this systematic review is described in detail elsewhere [34]. For drafting this systematic review, the checklist “Preferred Reporting Items for Systematic Reviews and Meta-Analyses: The PRISMA Statement” [35] (see Additional file 1) was utilized.

### Inclusion criteria

In this systematic review, (parallel-group or cluster-randomized) controlled trials (RCTs; CTs) were considered that described a school-based bicycle intervention fostering the use of bicycles in AST. Only samples that represented primary and/or secondary school students were included. The control group (CG) could be either active in terms of getting an alternative intervention program without strategies promoting AST or not receiving any kind of intervention. Only studies published in English and, due to current relevance, between 2000 and 2019 were included.

### Search strategy

A comprehensive search formula with a combination of keywords in three different categories according to “PICo” (population, intervention, context) [36] was generated in collaboration with two specialists (see Additional file 2). The first literature search based on title and abstract was conducted on November 28th, 2018 and was updated on November 25th, 2019 in eight electronic databases (ERIC: EBSCO, PsycINFO: EBSCO, PSYNDEX: EBSCO, PubMed: NCBI, Scopus: ELSEVIER, SPORTDiscus: EBSCO, SURF: BISp, and Web of Science: Clarivate Analytics).

### Study selection

**Fig. 1:**
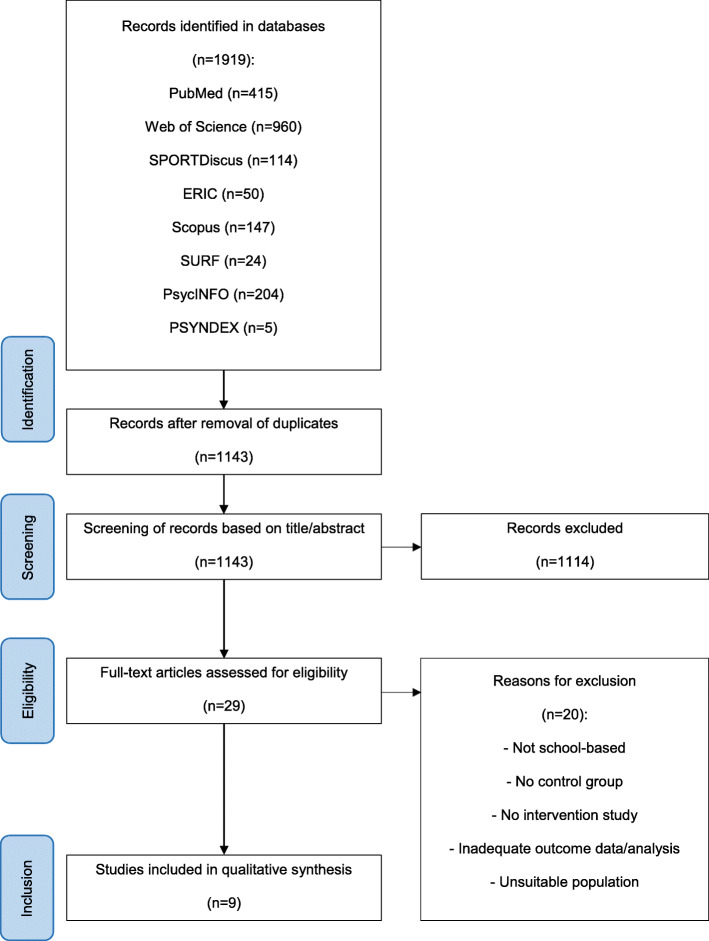
Procedure of study selection

Records were imported into and further managed with EndNote X7.4. The identified articles were screened independently by DS and TA/AM based on title, abstract, and full text in terms of their relevance and depicted in a flow chart (see Fig. 1). Any disagreements between the reviewers during these three steps of the selection process were resolved by discussion.

### Data extraction

Data regarding general study details, characteristics of participants, theoretical background, intervention description, outcome variables, measuring instruments, statistical analysis, and results were extracted using a previously piloted data extraction spreadsheet. Due to relevance, only intervention components that directly targeted AST were extracted. The authors of the included studies were contacted via e-mail with a maximum of two reminders if relevant data was missing or a clarification of descriptions was required. Therefore, the data extractors (DS and TA/AM) were not blinded to authors and journals while extracting study information. Two evaluators (DS and TA) independently coded the behavior change techniques (BCTs) applied to intervention components using the “BCT Taxonomy v1” [37]. Intervention components which could not be assigned to the 93 BCTs originally clustered in 16 main groups were classified into two newly added strategies/groups (i.e., knowledge transfer and parental involvement) by the authors according to a previously-used procedure [38]. Therefore, strategies were classified within a taxonomy of 95 BCTs clustered in 18 main groups. Any discrepancies were resolved by discussion.

### Quality assessment

For the assessment of the methodological quality of included studies, the quality assessment tool for quantitative studies “Effective Public Health Practice Project” (EPHPP) [39] was used. Where an explicit reference to a joint, more detailed article (e.g., study protocol) was mentioned, this article was additionally used to complete the assessment of the study’s methodological quality. Otherwise, articles in which the same intervention was analyzed with regard to different outcomes were assessed independently. A critical judgment was made for all items within the following eight quality sections/components (see Additional file 3): (A) Selection bias (two items), (B) Study design (four items), (C) Confounders (three items), (D) Blinding (two items), (E) Data collection methods (depending on the number of collected variables), (F) Withdrawals/Drop-outs (two items), (G) Intervention integrity (three items), and (H) Analyses (four items). Each item within the eight sections was assessed as strong, moderate or weak. The methodological quality of each item was rated independently by DS and TA/AM. Discrepancies between the evaluators despite discussions were resolved by consulting another independent evaluator (YD).

The following modifications to the EPHPP dictionary were made: Regarding the section (C) “confounders”, eight potentially relevant variables were chosen (i.e., age, sex/gender, previous AST experiences at baseline level, weight status, migration background, bicycle ownership, socio-economic status, distance from home to school) based on the “Model of Childrenʼs Active Travel” (M-CAT) [40]. When studies included five to eight of these potentially relevant variables as confounders, this item was rated as strong. It was rated as moderate when only three to four of potentially relevant variables were included and it was rated as weak when less than two of potentially relevant variables were included. In a further item of this section C, the quality of confounders was rated. If relevant to the study, the consideration of “sex/gender” [32] and “migration background” [31] led to a strong rating, whereas including “age” [32, 41] and “previous AST experience at baseline level” [42, 43] in the analysis were rated as moderate (weak: the rest). In the section (G) “intervention integrity”, the (unclear) presence of any kind of co-intervention or contamination led to a weak rating (strong: no co-intervention/contamination). Within the section (H) “analyses”, the item “unit of allocation” was rated as strong for “school”, as moderate for “class”, and as weak for “individual” based on the randomization level. The “unit of analysis” was appropriate and determined as strong when analyses were adjusted according to the “unit of allocation”. This means, for example, that analyses of a study cluster-randomized at school level had to be adjusted for schools. Otherwise, the “unit of analysis” was not appropriate and rated as weak.

After rating the individual items, each of the eight EPHPP quality components were assessed as a) strong (no weak ratings and more strong than moderate ratings), b) moderate (one weak rating), and c) weak (at least two weak ratings). Finally, a global quality rating based on the eight EPHPP components in each study was performed according to a common procedure [38]. When five or more components were assessed as strong and no components were assessed as weak, the global quality of a study was rated as strong [38]. The global quality of a study was rated as moderate when at least four components were assessed as strong and no more than one component was assessed as weak [38]. A weak methodological quality was rated when two or more components were assessed as weak [38].

### Data synthesis

All findings were summarized narratively by reporting effect sizes (ES), like Cohen’s d, Odds Ratio, partial Eta-squared (${\upeta}_{\mathrm{p}}^2$), and effect estimates, like confidence intervals (CI), or *p*-values (significant: *p* ≤ 0.05). The various outcome variables were grouped and studies were marked according to their effectiveness in terms of changing the related outcome(s). Results were sorted by age group (children up to the age of 12 years; adolescents from 13 years of age) [2]. If data allowed, gender effects were reported. Against our previous intention described in the published protocol [34], it was not possible to describe cultural dynamics based on regional differences. Given that only one outcome, i.e., body-mass-index (BMI), was considered in more than one intervention and measured/classified identically [44, 45], heterogeneity of variables across reviewed studies did not permit to carry out meta-analyses for intervention effects.

## Results

In total, 1711 publications were found in the first search and another 208 publications in the updated search. After removal of 776 duplicates, 1143 articles were screened. Nine relevant studies evaluating seven unique interventions were included in this review [44–52].

### Intervention characteristics and BCTs

The characteristics of the seven included interventions, evaluated in nine studies between 2012 and 2018, were heterogeneous (see Table 1). Interventions were carried out either in Europe (*n* = 5) or the USA (*n* = 2). Four interventions were designed as RCT. Only in three interventions, a sample size calculation was performed. Six out of seven interventions reported a sample size at baseline and indicated a range from 53 to 2401 participants. The number of recruited schools ranged from 1 to 25 (1 to 5 schools: *n* = 4; 14 schools: *n* = 1; 25 schools: *n* = 1). Primary schools and two grade levels were the most frequently chosen settings. The age of participants was up to 17 years (children: *n* = 4, children and adolescents: *n* = 3). Only five interventions reported the gender ratio of girls and boys. Interventions lasted between 4 weeks and 1 year and were classified into short-term (≤3 months: *n* = 3) or moderate-term (4 to 12 months: *n* = 2). Only one intervention included two different intervention arms (with/without parental involvement). Five interventions clearly stated that they did not deliver any kind of intervention to the CG. Three of these interventions, however, described either a provision of information (*n* = 1) or some kind of contamination in terms of minor interventions or similar conditions between the intervention group (IG) and CG (*n* = 2). Two interventions did not clearly report the conditions of the CG but mentioned contaminations, such as minor interventions, or delivery of informational letters. Three interventions reported that components were based on established theoretical frameworks, including the “Conceptual framework of AT in children”, the “Active Living by Design: 5P model” and the “Social Cognitive Theory”. One intervention was inspired by several correlates of cycling to school. In three interventions, no theoretical model was mentioned as a basis. The interventions included different components, such as a cycle training course or a bicycle train (i.e., adult-guided group of cycling children). Six interventions used a multicomponent approach with a combination of environmental, informational and behavioral (*n* = 2), environmental and informational (*n* = 1) or informational and behavioral (*n* = 3) components. One intervention was based on a behavioral approach only. Each intervention component was at least linked to one BCT. In total, 19 different applied BCTs were identified across the seven interventions.

**Table 1:** Intervention characteristics and strategies sorted by age group

Author, Year, Country, Design, Name of the Intervention	Participants	Theoretical Background	Intervention Description	Approach, Behavior Change Techniques [37]
Ducheyne et al., 2014 [47] Belgium Randomized controlled trial Not reported	Sample size determination: not reported *N* = 124 (cycling test)/114 (questionnaires) 4th grade students (3 primary schools); nIG(I) = 1; nIG(I + P) = 1; nCG = 1 Children aged 9 to 10 yrs	Not reported	IG(I): Master students provided a training course for basic cycling skills by using cycle games, practical cycling exercises et cetera on the school playground in a traffic-free environment during physical education for 4 wks (one 45 min session/wk). IG(I + P): After each session, wkly parental assisted homework tasks were provided (identify: 1. legal bike requirements, 2. the safest school cycling route, the most dangerous traffic spots close to the school, 3. if own bicycle considers legal requirements, 4. the correct meaning of different road signs). CG: No intervention.	Multicomponent (informational, behavioral): Social support (practical social support), shaping knowledge (instruction on how to perform the behavior, information about antecedents), comparison of behavior (demonstration of the behavior), repetition and substitution (behavioral practice/rehearsal)
Huang et al., 2018 [49] & Mendoza et al., 2017 [50] USA Randomized controlled trial Not reported	Sample size determination: GPower *N* = 54 4/5th grade students (4 primary schools); nIG = 24 (2); nCG = 30 (2) Nf = 64.8%, Nm = 35.2%; nIGf = 54.2%, nIGm = 45.8%; nCGf = 73.3%, nCGm = 26.7% Children aged 9 to 12 yrs. (9.9 ± 0.7 yrs); IG = 9.8 ± 0.8 yrs.; CG = 10.0 ± 0.7 yrs	Not reported	IG: For ca. 2 months (4 to 6 wks), daily provision of a voluntary bicycle train to/from school accompanied by study staff (duration: 10 to 45 min, school arrival: 25 to 30 min before start, school departure: 5 to 10 min after end time). Stops along the route were based on childrenʼs addresses to pick/drop them up/off. CG: No intervention but provision of usual “school transportation” information.	Behavioral: Shaping knowledge (instruction on how to perform the behavior), comparison of behavior (demonstration of the behavior), repetition and substitution (behavioral practice/rehearsal, behavior substitution, habit formation, habit reversal), antecedents (adding objects to the environment)
Østergaard et al., 2015 [45] Denmark Controlled trial “Tryg og Sikker Skolecykling” (Safe and secure cycling to school)	Sample size determination: not reported *N* = 2401 4/5th grade students (25 schools); nIG = 1296 (13); nCG = 1105 (12) nIGf = 48.9%, nIGm = 51.1%; nCGf = 51.2%, nCGm = 48.8% Children aged 9 to 11 yrs. (mean = 11 yrs); IG = 11.0 ± 0.64 yrs.; CG = 10.9 ± 0.63 yrs	Inspired by correlates of cycling to school (Hume et al., 2009; Timperio et al., 2006)	IG: The duration of the intervention was 1 yr. 1. Hard interventions implemented by local authorities at the school level (structural changes near the school, e.g., road surface, traffic regulation, signposting). 2. Soft interventions implemented by cycling federation at class level (cycling motivation, e.g., competitions and monitoring, and cycling safety, e.g., school traffic policy, cycle training and bicycle maintenance). Cycling incentives, e.g., school campaigns/events for parents/children, free helmets/gimmicks, were also provided. CG: No intervention but some minor interventions were still conducted in some schools.	Multicomponent (environmental, informational, behavioral): Feedback and monitoring (feedback on behavior), shaping knowledge (instruction on how to perform the behavior, information about antecedents), comparison of behavior (demonstration of the behavior, social comparison), repetition and substitution (behavioral practice/rehearsal), reward and threat (material incentive for behavior), antecedents (restructuring the physical environment, adding objects to the environment), knowledge transfer, parental involvement
Villa-González et al., 2015 [51], 2017 [52] Spain Controlled trial Not reported	Sample size determination: not reported *N* = 469 3rd to 5th grade students (5 primary schools); nIG = 295 (3); nCG = 174 (2) Nf = 46.5%, Nm = 53.5%; IGf = 47.8%, IGm = 52.2%; CGf = 44.3%, CGm = 55.7% Children aged 8 to 11 yrs	Conceptual framework of active travel in children (Panter et al., 2008)	IG: Teachers/researchers implemented monthly activities (each 60 to 120 min) in the classroom during regular school hours for 6 months (1. introduction with parental inclusion, e.g., mode of commuting survey and barriers, 2. story reading/performance of scenes related to AST) and school neighborhood (3. knowledge about environmental school characteristics, 4. road safety, 5. street behaviors, 6. AST and road safety education related traditional games). CG: No intervention.	Multicomponent (informational, behavioral): Shaping knowledge (instruction on how to perform the behavior, information about antecedents), comparison of behavior (demonstration of the behavior, social comparison, information about othersʼ approval), repetition and substitution (behavioral practice/rehearsal), parental involvement
Børrestad et al., 2012 [44] Norway Randomized controlled trial Active transportation to school and work in Norway	Sample size determination: yes *N* = 53 5th to 7th grade students (1 school); nIG = 26; nCG = 27 Nf = 47%, Nm = 53%; IGf = 46.1%, IGm = 53.9%; CGf = 48.1%, CGm = 51.9% Children/adolescents aged 10 to 13 yrs. (mean = 10.9 yrs); IG = 10.8 ± 0.7 yrs.; CG = 10.9 ± 0.7 yrs	Not reported	IG: For 12 wks, encouragement to cycle to/from school on a daily basis by providing six 30 min group sessions every second wk. during school hours (motivation by raising awareness, counteracting passive transport, parents support, health benefits from physical activity/cycling, road safety issues, cooperation with specialist in cycling safety). Provision of information and encouragement of cycling to school in parental sessions. Delivery of four parental informational letters (study aims/implications). Implementation by researchers/teachers. CG: Not reported but delivery of four parental informational letters (study aims/implications).	Multicomponent (informational, behavioral): Social support (unspecified social support), natural consequences (information about health consequences), knowledge transfer
Christiansen et al., 2014 [46] Denmark Randomized controlled trial SPACE–for physical activity	Sample size determination: not reported *N* = 1279 5/6th grade students (14 schools); nIG = 598 (7); nCG = 681 (7) IGf = 49%, IGm = 51%; CGf = 48.2%, CGm = 51.8% Children/adolescents aged 11.0 to 14.4 yrs.; IG/CG = 12.6 ± 0.63 yrs	Active Living by Design: 5P model (Bors et al., 2009)	IG: Eleven packages (four focused on AST). 1. Policy initiatives comprised a physical activity policy (reduction of school transport by car through parental encouragement to practice AST and be role models, acceptance of school traffic education initiatives and AST usage in educational settings, goal setting for AST and cooperation with municipalities/other stakeholders targeting environmental safety for AST). 2. Program initiatives consisted of a safe cycling education/training and a school traffic patrol (older students). 3. Physical initiatives included changes to enhance AST safety (e.g., cycle path, speed humps, new parking area, bike pool). 4. Preparation included a cross-disciplinary network (teachers, school leaders, municipality consultants, researchers). Awareness of AST benefits in students/parents. CG: Not reported but some minor interventions were already conducted in some schools.	Multicomponent (environmental, informational, behavioral): Goals and planning (action planning), social support (practical social support), shaping knowledge (instruction on how to perform the behavior), natural consequences (information about health consequences), comparison of behavior (demonstration of the behavior), repetition and substitution (behavioral practice/rehearsal, behavior substitution, habit formation, habit reversal), antecedents (restructuring the physical environment, adding objects to the environment)
Gutierrez et al., 2014 [48] USA Controlled trial Not reported	Sample size determination: GPower *N* = 58 intersections; nIG = 34 at 14 primary schools; nCG = 24 Children/adolescents aged 0 to 17 yrs	Social Cognitive Theory (Bandura, 1998)	IG: 1. Placement of 24 newly hired trained and equipped crossing guards. 2. Awareness campaigns done twice (presence/location via automated phone message for faculty/staff/parents, school specific location maps/safety information via handouts, school administration announcement for faculty/students/parents). CG: No intervention but identical crossing guard conditions.	Multicomponent (environmental, informational): Shaping knowledge (information about antecedents), antecedents (adding objects to the environment), parental involvement

*AST* active school travel, *ca.* circa, *CG* control group, *e.g.* for example, *f* female, *I(G)* intervention (group), *m* male, *min* minute(s), *N* total sample size, *n* subgroup sample size, *P* parents, *wk./ly/s* week/ly/s, *yr(s)* year(s)

These 19 different applied BCTs were clustered in a total of 11 out of 18 main groups (see Table 2), which varied in their popularity: (1) Shaping knowledge (*n* = 6), (2) Comparison of behavior (*n* = 5), (3) Repetition and substitution (*n* = 5), (4) Antecedents (*n* = 4), (5) Social support (*n* = 3), (6) Parental involvement (*n* = 3), (7) Natural consequences (*n* = 2), (8) Knowledge transfer (*n* = 2), (9) Feedback and monitoring (*n* = 1), (10) Reward and threat (*n* = 1), (11) Goals and planning (*n* = 1). The seven interventions used in average 4.7 main groups.

**Table 2:**
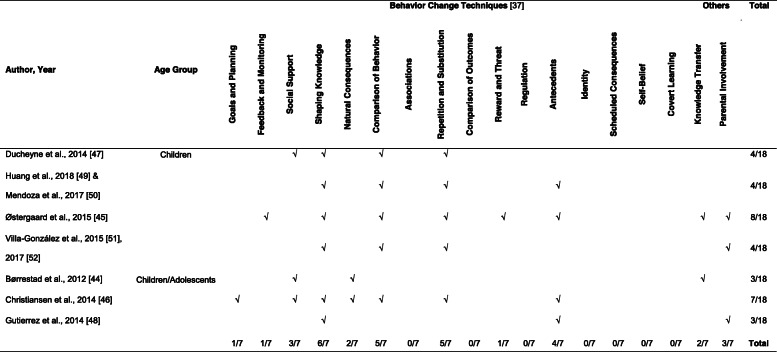
Applied behavior change techniques in reviewed interventions sorted by age group

### Study quality

All included studies were assessed as weak in the global rating but none of the nine studies had a weak rating in all eight sections (see Table 3).

**Table 3:** Sectional and global quality rating of reviewed studies sorted by age group

Author, Year	Age Group	Sectional Rating								Global Rating
		Selection Bias	Study Design	Confounders	Blinding	Data Collection Methods	Withdrawals/Drop-Outs	Intervention Integrity	Analyses	
Ducheyne et al., 2014 [47]	Children	Moderate	Strong	Weak	Weak	Moderate	Moderate	Weak	Weak	Weak
Huang et al., 2018 [49]		Weak		Moderate	Weak	Strong	Moderate		Strong	Weak
Mendoza et al., 2017 [50]		Moderate		Moderate	Weak	Strong	Strong		Strong	Weak
Østergaard et al., 2015 [45]		Moderate		Strong	Weak	Weak	Moderate		Weak	Weak
Villa-González et al., 2015 [51]		Moderate		Strong	Weak	Weak	Moderate		Weak	Weak
Villa-González et al., 2017 [52]		Moderate		Moderate	Weak	Moderate	Moderate		Strong	Weak
Børrestad et al., 2012 [44]	Children/Adolescents	Weak		Strong	Moderate	Strong	Strong		Weak	Weak
Christiansen et al., 2014 [46]		Moderate		Moderate	Weak	Weak	Moderate		Strong	Weak
Gutierrez et al., 2014 [48]		Moderate		Weak	Weak	Moderate	Weak		Weak	Weak

**Fig. 2:**
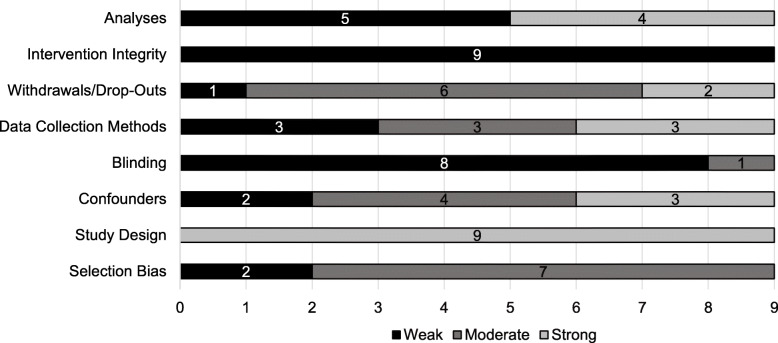
Quality rating of sections across reviewed studies

Figure 2 gives an overview of the study quality for individual sections across all reviewed studies. Due to the inclusion of RCTs and CTs only, the section with the strongest methodological quality was “study design” rated as strong in all nine studies. Additional strong ratings were found in the sections “confounders”, “data collection methods”, “withdrawals/drop-outs”, and “analyses”. In the section “confounders”, only one study [48] did not report adjustments. The other eight studies [44–47, 49–52] reported adjustments for at least two up to eight out of ten different covariates (i.e., age, distance from home to school, sex/gender, AST, BMI, race, bike score, neighbourhood disorder, attendance, accelerometer wear time). However, group differences at baseline were only absent in two studies [44, 51]. In the section “data collection methods”, three studies were rated as weak [45, 46, 51], three as moderate [47, 48, 52], and three as strong [44, 49, 50]. Referring to the section “withdrawals/drop-outs”, six studies declared drop-outs [44, 46, 47, 50–52] and five studies had low retention rates [45, 48, 49, 51, 52]. The sections “selection bias” and “blinding” were never rated as strong. Apart from two studies [46, 48], seven studies did not report the representativeness of the sample. Six studies [45–47, 50–52] reached a high recruitment rate. All but one study [44] either did not report blinding at all or reported unblinded conditions. In the section “analyses” ratings were either strong [46, 49, 50, 52] or weak [44, 45, 47, 48, 51] with strengths in the unit of allocation [45–47, 49–52] as well as statistical methods (including ES) [44–46, 48–50, 52] and deficits in the unit of analyses [45, 47, 48, 51] as well as usage of intention-to-treat analysis [44, 45, 47, 48, 51].

The weakest section was “intervention integrity” rated as weak in all nine studies. Only one study [45] indicated the percentage of intervention delivery and measurement of consistency. Moreover, six out of nine studies [44–46, 48–50] described a potential contamination in the CG.

### Intervention effects

Altogether, six studies reported proportionally more non-significant than significant intervention effects [44–48, 51]. One study found more adverse intervention effects in boys with larger improvements in the CG [52]. Only two studies – describing the same intervention in children: a “bicycle train” to actively travel to school – showed significant beneficial intervention effects in all their seven outcomes [49, 50] (see Tables 4 and 5).

**Table 4:** Outcome variables, measuring instruments, covariates and intervention effects in reviewed studies sorted by age group

Author, Year	Age Group	Outcomes (Measuring Instruments)	Adj. for Covariates	Intervention Effects
Ducheyne et al., 2014 [47]	Children	Cycling skills (objective: practical cycling test); AST: min of cycling to school (subjective: questionnaire); Psychosocial factors: attitudes towards cycling (subjective: questionnaire)	Baseline values of age, distance from home to school	Total basic cycling skills (adj.): group difference from pre to post, pre to follow-up and pre to post to follow-up with greater increase in IG(I/I + P) (*p* < 0.001, respectively), n.s. group difference from post to follow-up with greater increase in CG Min of cycling to school last wk. (adj.): n.s. group difference from pre to post, pre to follow-up and pre to post to follow-up, group difference from post to follow-up with decrease in IG(I/I + P) and increase in CG (*p* < 0.05) Parental attitudes towards cycling (adj.): n.s. group difference at any time point in importance/encouragement of cycling to school, importance of cycling skills/cycle training, encouragement of cycling skills improvement, impact of cycling training course on safer cycling behaviors in real traffic situations, and feeling of safety when cycling in traffic
Huang et al., 2018 [49]		Psychosocial factors: self-efficacy, outcome expectations (subjective: questionnaire)	Race/ethnicity, age, BMI z-score, bike score, sex/ gender, neighborhood disorder, distance from home to school	Child self-efficacy (adj.): group difference from pre to post (*p* < 0.05; β = 0.84 [CI95: 0.37, 1.31]) with increase in IG (β = 0.40 [CI95: 0.05, 0.75]) and decrease in CG (*p* < 0.05; β = − 0.43 [CI95: − 0.76, −0.11]) Parental self-efficacy (adj.): group difference from pre to post (*p* < 0.05; β = 0.46 [CI95: 0.05, 0.86]) with increase in IG (β = 0.21 [CI95: − 0.09, 0.51]) and decrease in CG (n.s.; β = − 0.25 [CI95: − 0.52, 0.03]) Parental outcome expectations (adj.): group difference from pre to post (*p* < 0.05; β = 0.47 [CI95: 0.17, 0.76]) with increase in IG (β = 0.14 [CI95: − 0.07, 0.36]) and decrease in CG (*p* < 0.05; β = − 0.32 [CI95: − 0.52, − 0.12])
Mendoza et al., 2017 [50]		AST: % of daily cycling trips to school (subjective: questionnaire); PA levels: MVPA (total, cycling, before/after school) in av. min/day (objective: accelerometer, GPS units)	Race/ethnicity, age, bike score, BMI z-score, sex/gender, neighborhood disorder, distance from home to school, accelerometer wear time	% of daily cycling trips to school (adj.): group difference from pre to post (*p* < 0.05; β = 44.9 [CI95: 26.8, 63.0]) with greater increase in IG (n.s.; β = 0.10 [CI95: − 0.02, 0.23]) Total MVPA in av. min/day (adj.): group difference from pre to post (*p* < 0.05; β = 21.6 [CI95: 8.7, 34.6]) with increase in IG and decrease in CG (n.s.; β = − 4.8 [CI95: − 13.6, 4.0]) Cycling MVPA in av. min/day (adj.): group difference from pre to post (*p* < 0.05; β = 23.0 [CI95: 10.7, 35.4]) with decrease in CG (n.s.; β = − 1.6 [CI95: − 10.0, 6.8]) Before/after school MVPA in av. min/day (adj.): group difference from pre to post (*p* < 0.05; β = 12.8 [CI95: 8.5, 17.2]) with decrease in CG (n.s.; β = − 0.9 [CI95: − 3.8, 2.1])
Østergaard et al., 2015 [45]		PA levels: LTPA beyond AST (subjective: questionnaire); AST: frequency of long/short-term school cycling (trips) (subjective: questionnaire); AT: frequency of cycling beyond school (subjective: questionnaire); Physical fitness (CRF): aerobic capacity (objective: Andersen test); Weight status: BMI (objective: digital scale, stadiometer)	Age, baseline BMI, baseline value, sex/ gender	LTPA beyond AST (adj.): n.s. group difference from pre to post with decrease in IG (β = − 0.09 [CI95: − 0.21, 0.03]) Frequency of long-term school cycling (adj.): n.s. group difference from pre to post with decrease in IG (β = − 0.02 [CI95: − 0.10, 0.05]) Frequency of short-term school cycling trips last wk. (adj.): n.s. group difference from pre to post with increase in IG (β = 0.15 [CI95: − 0.25, 0.54]) Frequency of cycling beyond school last wk. (adj.): n.s. group difference from pre to post with decrease in IG (β = − 0.04 [CI95: − 0.14, 0.05]) Aerobic capacity (adj.): group difference from pre to post with decrease in IG (*p* < 0.001; β = − 1.45 [CI95: − 1.92, − 1.00]) BMI (adj.): n.s. group difference from pre to post with increase in IG (β = 0.01 [CI95: − 0.13, 0.15]) Risk of developing overweight/obesity (adj.): n.s. group difference from pre to post with increase in IG (OR = 0.88 [CI95: 0.50, 1.57]) Dose response association between cycling to school and total intensity (adj.): n.s.
Villa-González et al., 2015 [51]		AST: mode/frequency of (active) trips to school (subjective: questionnaire)	Sex/gender, age, distance from home to school, pre/post AST variables, attendance	Mode of trips to school last wk. (adj.): n.s. group difference from pre to post in walking with greater increase in CG and biking with decrease in CG and no change in IG, group difference from post to follow-up in walking only with increase in IG and decrease in CG (*p* = 0.004) Frequency of active trips to school last wk. (adj.): n.s. group difference from pre to post in walking and cycling with greater increase in CG, group difference from post to follow-up in walking and cycling with increase in IG and decrease in CG (*p* = 0.019)
Villa-González et al., 2017 [52]		AST: mode/frequency of (active) trips to school (subjective: questionnaire); Physical fitness: CRF (VO_2max_, 20-m shuttle run test), muscular fitness (standing long jump, handgrip strength), speed agility (4 × 10 shuttle run test) (objective: ALPHA health-related fitness test battery)	Age, distance	Mode of trips to school last wk. (adj.): n.s. group difference from pre to post in walking, group difference from pre to post in cycling with increase in IG for male only and decrease in CG for male (*p* = 0.04) Frequency of active trips to school last wk. (adj.): n.s. group difference from pre to post CRF (adj.): group difference from pre to post in VO_2max_ with increase in CG for male only and decrease in IG for male and 20-m shuttle run test with increase in CG for male only and no change in IG for male (*p* < 0.05, respectively) Muscular fitness (adj.): n.s. group difference from pre to post in standing long jump, group difference in handgrip strength with increase in CG in male only and decrease in IG for male (*p* < 0.05) Speed agility (adj.): n.s. group difference from pre to post in 4 × 10 shuttle run test
Børrestad et al., 2012 [44]	Children/ Adolescents	Physical fitness (CRF): VO_2peak_ (objective: cycle ergometer), HR_peak_ (objective: heart rate monitor); Weight status: BMI, overweight (objective: beam scale, stationmeter); AST: start cycling (subjective: questionnaire)	Baseline level, sex/gender, age	VO_2peak_ (adj.): n.s. group difference from pre to post (d = − 0.13) with increase in IG [CI95: 47.5, 51.8] and CG [CI95: 48.5, 52.8] HR_peak_ (adj.): n.s. group difference from pre to post (d = 0.03) with increase in IG [CI95: 189.4, 197.5] and decrease in CG [CI95: 189.2, 197.2] BMI (adj.): n.s. group difference from pre to post (d = 0.01) with no change in IG [CI95: 18.5, 19.1] and increase in CG [CI95: 18.3, 13.9] Overweight (adj.): n.s. group difference from pre to post with decrease in IG [CI95: 8.0, 33.7] and increase in CG [CI95: 7.7, 34.6] Start cycling last 3 mos: n.s. group difference from pre to post with greater increase in IG [CI95: 50.1, 88.2] than CG [CI95: 20.9, 60.5]
Christiansen et al., 2014 [46]		AST: total no. of active trips to school (subjective: transport diary); Psychosocial factors: perceived route safety to school, encouragement of cycling to school, attitude towards cycling (subjective: questionnaire)	Age, baseline proportion of AST, distance to school, sex/ gender	Total no. of active trips to school for previous day over 5 days (adj.): n.s. group difference from pre to post with increase in IG and CG (OR = 1.27 [CI95: 0.81, 1.99]), n.s. gender effect with increase in male in IG and CG Perceived route safety to school of student (adj.): n.s. group difference from pre to post with decrease in IG and increase in CG (OR = 0.87 [CI95: 0.50, 1.51]) Parental encouragement of cycling to school (adj.): n.s. group difference from pre to post with increase in IG and CG (OR = 1.26 [CI95: 0.92, 1.73]) Student attitude towards cycling (adj.): n.s. group difference from pre to post with decrease in IG and CG (OR = 1.50, [CI95: 0.90, 2.50])
Gutierrez et al., 2014 [48]		AST: counts of intersection crossings (objective: observation); Psychosocial factors: perception of safety, attitudes/beliefs towards AST (subjective: questionnaire)	NR	Counts of intersection crossings: n.s. group difference from pre to post in AST trends with increase in IG and CG, n.s. between-intersection effects from pre to post in no. of crossing guards (${\upeta}_{\mathrm{p}}^2$ = 0.00), experimental intersections (${\upeta}_{\mathrm{p}}^2$ = 0.00) or interaction of experimental and supervised intersections (${\upeta}_{\mathrm{p}}^2$ = 0.01), increase in usage of supervised intersections in IG and CG (*p* = 0.041; ${\upeta}_{\mathrm{p}}^2$ = 0.08) Parental perception of safety: no change (n.s.) Parental attitudes/beliefs towards AST**:** no change (n.s.)

*adj.* adjustment/adjusted, *AST* active school travel, *AT* active travel, *av.* average, *β* beta coefficient, *BMI* body-mass-index, *CG* control group, *CI* confidence interval, *CRF* cardiorespiratory fitness, *d* effect size (Cohen), *GPS* Global Positioning System, *HR*_*peak*_ peak heart rate, *I(G)* intervention (group), *LTPA* leisure-time physical activity, *m* meter, *min* minute(s), *mos* months, *MVPA* moderate-to-vigorous physical activity, *no.* number, *NR* not reported, *n.s.* not significant, *OR* Odds Ratio, *P* parent, *p* probability value, *PA* physical activity, *VO*_*2max*_ maximal oxygen uptake, *VO*_*2peak*_ peak oxygen uptake, *wk.* week, ${\upeta}_{\mathrm{p}}^2$ partial Eta-squared

**Table 5:** Overview of outcome variables and intervention effects across reviewed studies sorted by age group

Outcome variables				Intervention effects (Pre/Post)	
				Children	Children/Adolescents
**AST**	**Subjective**	Mode of trips to school		0^51^_a_, +m^52^_a_	
		Total no. of active trips to school			0^46^
		Frequency of active trips to school		0^51^_a_, 0^52^_a_	
		Frequency of long/short-term school cycling (trips)		0^45^, 0^45^	
		% of daily cycling trips to school		+^50^_b_	
		Min of cycling to school		0^47^	
		Start cycling			0^44^
	**Objective**	Counts of intersection crossings			0^48^
**Psychosocial Factors**	**Subjective**	Parental attitude/beliefs towards AST			0^48^
		Parental attitudes towards cycling		0^47^	
		Student attitude towards cycling			0^46^
		Parental perception of safety			0^48^
		Perceived route safety to school of student			0^46^
		Parental encouragement of cycling to school			0^46^
		Parental/Child self-efficacy		+^49^_b_/+^49^_b_	
		Parental outcome expectations		+^49^_b_	
**Physical Fitness**	**Objective**	**CRF**	Aerobic capacity	-^45^	
			VO_2peak_		0^44^
			HR_peak_		0^44^
			VO_2max_	+CGm^52^_a_	
			20-m shuttle run test	+CGm^52^_a_	
		**Muscular Fitness**	Standing long jump	0^52^_a_	
			Handgrip strength	+CGm^52^_a_	
		**Speed Agility**	4x10 shuttle run test	0^52^_a_	
**PA Levels**	**Subjective**	LTPA beyond AST		0^45^	
	**Objective**	MVPA (total, from cycling, before/after school) in av. min/d		+^50^_b_/+^50^_b_/+^50^_b_	
**Weight Status**	**Objective**	BMI		0^45^	0^44^
		Overweight			0^44^
		Risk of developing overweight/obesity		0^45^	
**AT**	**Subjective**	Frequency of cycling beyond school		0^45^	
**Cycling Skills**	**Objective**	Total basic cycling skills		+^47^	

Note: The symbol + indicates an intervention effect, - marks unfavorable intervention effects in the intervention condition, and 0 means no intervention effect. The letters CG declare intervention effects in favor of the control condition. The letter m depicts intervention effects in favor of males. The letters _a_/_b_ indicate studies with the same intervention, respectively

*AST* active school travel, *AT* active travel, *av.* average, *BMI* body-mass-index, *CRF* cardiorespiratory fitness, *d* day, *HR*_*peak*_ peak heart rate, *LTPA* leisure-time physical activity, *m* meter, *min* minute(s), *MVPA* moderate-to-vigorous physical activity, *no.* number, *PA* physical activity, *VO*_*2max*_ maximal oxygen uptake, *VO*_*2peak*_ peak oxygen uptake

In total, 35 different outcome variables were reported across the nine included studies. These 35 outcome variables were clustered in seven main outcome groups: (1) AST (*n* = 9), (2) Psychosocial factors targeting both parents or students (*n* = 9), (3) Physical fitness divided into cardiorespiratory/muscular fitness and speed agility (*n* = 8), (4) PA levels (*n* = 4), (5) Weight status (*n* = 3), (6) AT (*n* = 1), and (7) Cycling skills (*n* = 1).

A significant intervention effect was found on 13 different outcomes analyzed across five studies [45, 47, 49, 50, 52], whereas seven studies reported non-significant effects on 25 outcomes in total [44–48, 51, 52]. Within the outcome group “AST”, one study found a significant beneficial intervention effect on bicycle trips to school by boys [52] and another study on percentage of daily cycling trips to school (β = 44.9 [CI95: 26.8, 63.0]) [50]. One study, investigating psychosocial factors only, showed significant beneficial intervention effects on parental (β = 0.46 [CI95: 0.05, 0.86]) and child self-efficacy (β = 0.84 [CI95: 0.37, 1.31]) as well as parental outcome expectations (β = 0.47 [CI95: 0.17, 0.76]) [49]. Within the outcome group “physical fitness”, one study found a significant adverse intervention effect on aerobic capacity with an unfavorable development in the IG (β = − 1.45 [CI95: − 1.92, − 1.00]) [45]. Another study found significantly higher values in the CG for boys only on VO_2max_ (group main effect: ${\eta}_{\mathrm{p}}^2=0.01$), 20-m shuttle run test (group main effect: ${\eta}_{\mathrm{p}}^2=0.04$), and handgrip strength [52]. Within the outcome group “PA levels”, one study reported positive intervention effects on total MVPA (β = 21.6 [CI95: 8.7, 34.6]), MVPA from cycling (β = 23.0 [CI95: 10.7, 35.4]) and MVPA before/after school (β = 12.8 [CI95: 8.5, 17.2]) [50]. One study found a significant intervention effect on total basic cycling skills in both intervention arms (with/without parental involvement), which were taken together in this analysis [47].

The sustainability of intervention effects were examined in only two studies at 5- [47] or 6-month follow-up [51]. After participating in a 4-week cycle training course, a significant intervention effect from pre to post to follow-up for both intervention arms (with/without parental involvement) was found on total basic cycling skills but neither on cycling to school (in min) nor on parental attitudes towards cycling [47]. Significant effects at 6-month follow-up were found on “mode of trips to school” in walking only and “frequency of active trips to school” (walking/cycling) even though non-significant intervention effects from pre to post after 6 months were shown on these variables [51].

## Discussion

The aims of this systematic review were to provide an overview of existing school-based interventions focusing on the promotion of AST by bicycle in children and adolescents and their evidence on strategies and effects. Following our inclusion criterion for study designs, we only found a small number of (R)CTs in our literature search. This is consistent with the reported gap of strong study designs in this research field [53]. The included trials were predominantly not conducted in cycle-centric countries within Europe (exception: Belgium (*n* = 1) and Denmark (*n* = 2)) [54], showed a large variety of components and outcome measures, and were of weak quality. Three of the included trials did not differentiate between walking and cycling as two different types of AST in their analyses [46, 48, 51, 52]. Therefore, a final conclusion on cycling to school could not be drawn from these studies. Additionally, the reported interventions were designed for children only or both children and adolescents, implemented in primary schools. The lack of interventions for adolescents, implemented in secondary schools, is also in line with the current state of research [55]. In conclusion, the findings of our systematic review need to be interpreted with caution.

### Promising intervention strategies

Overall, only one intervention using a single-component approach showed consistent positive effects on all measured outcome variables [49, 50] and provides first insights into an effective intervention strategy. For approximately 2 months, a voluntary and adult-guided bicycle train to/from school with pick up/drop off stops was provided for children on schooldays [49, 50] including the following main groups of BCTs: shaping knowledge, comparison of behavior, repetition and substitution as well as antecedents. The counterpart of a bicycle train, the “walking school bus” (WSB), is based on a similar approach for walking. In a previous review, the WSB was found to increase walking to school as well as general PA levels in children [24]. However, the bicycle train intervention effect on MVPA from cycling (23.0 min/day) was higher than the intervention effect on total MVPA (21.6 min/day) [50]. This accelerometer data might suggest a compensation in total MVPA due to the additional MVPA from AST by bicycle.

The only study that performed a sex/gender analysis reported increased bicycle trips to school in boys but not in girls [52]. As boys had higher levels of health-related fitness than girls despite comparable low cycling to school rates at baseline [52], poor fitness could be a barrier to uptake AST by bicycle in girls. More research on the existence and explanation of gender differences in intervention effects is warranted in future studies to draw final conclusions.

### Strengths/limitations

The major strengths of this systematic review are the specific focus on school-based interventions that promote cycling to school and including only (R)CTs, which provide a higher evidence level than other study designs [56]. Two researchers independently conducted the process of selecting studies, extracting data, evaluating methodological quality and BCTs. Furthermore, authors of included studies were contacted in case of missing data to avoid an underestimation of the methodological quality. Finally, findings were interpreted separately from the methodological quality rating in order to provide transparency.

A limitation is that the defined criterion of including only (R)CTs could have led to a selection bias [53]. The same applies to the restriction of studies published in English. At study level, there are several reasons for a lack of effectiveness. One reason could be the complete absence of intervention periods longer than 13 months [57]. According to the “Transtheoretical Model of Behavior Change”, “individuals may need to go through a number of stages associated with the formulation and implementation of attitudes and beliefs before actually undertaking changes, and this whole process takes some time” [58] (p. 68). This is why a lack of immediate success in short- or moderate-term interventions might not necessarily indicate a failure of the intervention [59]. The adoption and integration of cycling to school into the daily routine could have happened after the observed period. Another reason for a lack of effectiveness could be that different local needs in terms of barriers to cycle to school were not sufficiently addressed in interventions [60]. In a previous study, barriers of AST in general were categorized according to the “Social-ecological model of the correlates of AT” [61]: intrapersonal/individual (i.e., child factors), interpersonal (e.g., parental factors), community (e.g., school policy), and environment (e.g., traffic) [62]. One multicomponent intervention among children was inspired by correlates of cycling to school considering such barriers (e.g., intrapersonal/individual including motivation by competitions and safety by cycle training, interpersonal including parental involvement, community including school policies, and environmental changes including traffic regulation) and used almost the same BCTs as the effective bicycle train intervention (apart from repetition and substitution including behavior substitution, habit formation, habit reversal) [45]. Despite this, this intervention was not effective on any outcome in favor of the IG [45]. Furthermore, the improvement of basic cycling skills in a cycle training program among children (examined in only one intervention) without practicing traffic-related skills in the natural environment may be insufficient to impact AST by bicycle [47]. Moreover, a cycle training program including parental assisted homework tasks (e.g., identification of the safest school cycling route and the most dangerous traffic spots close to the school) after each cycle training failed to find effective ways of involving parents as an intervention strategy [47]. The reason could be that the homework tasks insufficiently addressed or increased personal safety barriers in parents (e.g., fears, dangers, concerns about the child’s behavior in road traffic) [62]. This may have blocked behavior change in their child as the influence of parents on AST is higher among children than adolescents [40]. Therefore, adolescents may need different intervention strategies than children because all five studies that effectively influenced 13 of 24 examined outcomes included children only [45, 47, 49, 50, 52] and three of the four studies that were not effective in influencing any outcome included both children as well as adolescents [44, 46, 48]. To adequately tailor interventions to a specific population, we recommend following a systematic approach when developing interventions (e.g., the “Intervention Mapping Approach” including a comprehensive needs assessment and theoretical frameworks [63]). Moreover, we recommend conducting a process evaluation that provides insights into the implementation of the intervention (e.g., feedback on program and material, (dis)satisfaction). In addition, we recommend using a checklist when reporting the study. Adherence to the planned intervention (e.g., delivered intensity) was lacking in the majority of studies although “the dose of an intervention is a key predictor of behavior change” [58] (p. 68). Furthermore, contamination was quite common and could have caused an underestimation of effects. Finally, interpretations of findings could be biased due to group differences at baseline.

## Conclusions

As a result of the heterogeneity and low methodological quality of included studies, we conclude that the evidence for the effectiveness of interventions promoting AST by bicycle is insufficient. Therewith, our findings confirm that this research field is still in an early development stage [57]. Nevertheless, there is an indication that a bicycle train to/from school among children in primary school, including four clustered main groups of BCTs (shaping knowledge, comparison of behavior, repetition and substitution as well as antecedents), is a promising intervention. More research is needed to better understand strategies and effects of school-based interventions promoting AST by bicycle, especially among adolescents in secondary school.

Based on the findings of this systematic review, there is a need for high-quality intervention studies in this research field. This is why future studies are recommended to evaluate theory-based interventions in longer-term (R)CTs using relevant, valid and reliable outcome measures. Additionally, more research is warranted to examine the moderating effect of gender in AST interventions by bicycle and to prove long-term maintenance of behavior change.

## Supplementary information

**Additional file 1.** Preferred Reporting Items for Systematic Reviews and Meta-Analyses: The PRISMA Statement.

**Additional file 2.** Search formula used in the eight electronic databases.

**Additional file 3.** Sections, components and items of the quality assessment tool.

## Data Availability

Not applicable.

## Abbreviations

AST: Active school travel
AT: Active transport
β: Beta coefficient
BCT(s): Behavior change technique(s)
BMI: Body-mass-index
CG: Control group
CI: Confidence interval
CT(s): Controlled trial(s)
e.g.: For example
EPHPP: Effective Public Health Practice Project
ES: Effect size(s)
h: hour(s)
i.e.: That is
IG: Intervention group
m: Meter(s)
M-CAT: Model of Childrenʼs Active Travel
min: Minute(s)
MVPA: Moderate-to-vigorous intensity physical activity
n: Number(s)
p: Probability value
PA: Physical activity
PICo: Population, interest, context
RCT(s): Randomized controlled trial(s)
VO_2_max: Maximal oxygen uptake
WHO: World Health Organization
WSB: Walking school bus
