# Supplementary material for: Strategies and effects of school-based interventions to promote active school transportation by bicycle among children and adolescents: a systematic review
Source: Int J Behav Nutr Phys Act. 2020 Nov 12;17:138. doi: 10.1186/s12966-020-01035-1 (PMC7661215; doi:10.1186/s12966-020-01035-1)
Supplement: Supplementary file 2 — Additional file 2. Search formula used in the eight electronic databases. [file 12966_2020_1035_MOESM2_ESM.docx]

Additional file 2. Search formula used in the eight electronic databases.

|  | **Search Terms** |
| --- | --- |
| 1. | (child*) |
| 2. | (infan*) |
| 3. | (adolescen*) |
| 4. | (preadolescent) |
| 5. | (juven*) |
| 6. | (teen*) |
| 7. | (young*) |
| 8. | (youth*) |
| 9. | (student*) |
| 10. | (pupil*) |
| 11. | (boy*) |
| 12. | (girl*) |
| 13. | (kid*) |
| 14. | (pediatric*) |
| 15. | (intervention*) |
| 16. | (training*) |
| 17. | (experiment*) |
| 18. | (program*) |
| 19. | (education*) |
| 20. | (treatment*) |
| 21. | (evaluat*) |
| 22. | (course*) |
| 23. | (outcome*) |
| 24. | (effect*) |
| 25. | (result*) |
| 26. | (evidence) |
| 27. | (finding*) |
| 28. | (impact*) |
| 29. | (encourag*) |
| 30. | (improv*) |
| 31. | (increas*) |
| 32. | (promot*) |
| 33. | (enhanc*) |
| 34. | (travel*) |
| 35. | (transport*) |
| 36. | (commut*) |
| 37. | (cycl*) |
| 38. | (bicycl*) |
| 39. | (bik*) |
| 40. | (school*) |
| 41. | (class*) |
| 42. | (physical education) |
| 43. | (lesson*) |
| 44. | 1 OR 2 OR 3 OR 4 OR 5 OR 6 OR 7 OR 8 OR 9 OR 10 OR 11 OR 12 OR 13 OR 14 |
| 45. | 15 OR 16 OR 17 OR 18 OR 19 OR 20 OR 21 OR 22 OR 23 OR 24 OR 25 OR 26 OR 27 OR 28 OR 29 OR 30 OR 31 OR 32 OR 33 |
| 46. | 34 OR 35 OR 36 |
| 47. | 37 OR 38 OR 39 |
| 48. | 40 OR 41 OR 42 OR 43 |
| 49. | 44 AND 45 AND 46 AND 47 AND 48 |
| 50. | Removal of duplicates from 49 |
