# Supplementary material for: Strategies and effects of school-based interventions to promote active school transportation by bicycle among children and adolescents: a systematic review
Source: Int J Behav Nutr Phys Act. 2020 Nov 12;17:138. doi: 10.1186/s12966-020-01035-1 (PMC7661215; doi:10.1186/s12966-020-01035-1)
Supplement: Supplementary file 3 — Additional file 3. Sections, components and items of the quality assessment tool. [file 12966_2020_1035_MOESM3_ESM.docx]

Additional file 3. Sections, components and items of the quality assessment tool.

| **Section** | **Component** | **Items** |
| --- | --- | --- |
| A | Selection Bias | Representativeness of sample; Percentage of recruitment rate |
| B | Study Design | RCT or CT; Randomization; Method of randomization; Appropriateness of randomization method |
| C | Confounders | Group differences prior to intervention; Relevant confounders according to the “Model of Childrenʼs Active Travel” (40): age, sex/gender, previous AST experiences at baseline level, weight status, migration background, bicycle ownership, socio-economic status, distance from home to school; Quality rating of controlled confounders |
| D | Blinding | Blinding of outcome assessor(s); Blinding of participants |
| E | Data Collection Methods | Validity of data collection tools; Reliability of data collection tools |
| F | Withdrawals/ Drop-Outs | Report of drop-outs (numbers/reasons); Percentage of retention rate |
| G | Intervention Integrity | Percentage of intervention delivery; Measurement of interventionʼs consistency; Contamination/co-intervention |
| H | Analyses | Unit of allocation; Unit of analysis; Appropriateness of statistical methods; Intention to treat |

AST=active school travel; CT=controlled trial; RCT=randomized controlled trial
